# Hemifacial spasm is not affected by state of consciousness: a case report

**DOI:** 10.1186/s40001-021-00616-5

**Published:** 2021-12-07

**Authors:** Tao Li, Zhuo Feng, Chunli Song, Zhanhua Liang

**Affiliations:** 1 Department of Neurology, First Affiliated Hospital of Dalian Medical University, Dalian, China; 2 Department of Emergency, First Affiliated Hospital of Dalian Medical University, Dalian, China; 3 Department of Neuroelectrophysiology and Botulinum Toxin Clinic, First Affiliated Hospital of Dalian Medical University, Dalian, China

**Keywords:** Hemifacial spasm, Coma, Differential diagnosis, Intensive care unit

## Abstract

**Background:**

Hemifacial spasm (HFS) is a movement disorder caused by mechanical compression of the facial nerve after it has left the brainstem and is characterized by brief or sustained twitching of the muscles innervated by that nerve. Often we observe spasm in an awakening situation. Actually contractions persist during sleep. To our knowledge, there were no reports on how HFS manifests under disturbance of consciousness. Here, we report a case of primary HFS in which the patient's symptoms persisted in a coma.

**Case presentation:**

A 74-year-old female with right-sided primary HFS for 20 years and had received botulinum toxin injections in our hospital. Unfortunately she was carried to emergency department after traumatic right pneumothorax by accident. During the emergency treatment, she lost consciousness due to simultaneous cardiac arrest and respiratory arrest. She was then admitted to the emergency intensive care unit for further treatment. During her hospitalization, she was in a coma with stable vital signs and persisting symptoms of HFS. Thus, a multidisciplinary consultation was requested to identify whether it was focal cortical seizures involving the right-side facial muscles. Physical examination revealed brief involuntary clonic or tonic contractions accompanied with the ‘Babinski-2 sign’. A combination of relevant data, including her past history, clinical presentation and a negative computed tomography scan of the head, led to a diagnosis of right-sided HFS. As the symptoms of HFS are not life-threatening, the use of anticonvulsants is unnecessary.

**Conclusions:**

For the layperson, it is crucial to seek a multidisciplinary consultation to obtain a correct diagnosis.

**Supplementary Information:**

The online version contains supplementary material available at 10.1186/s40001-021-00616-5.

## Background

Hemifacial spasm (HFS) is a movement disorder caused by mechanical compression of the facial nerve after it has left the brainstem and is characterized by brief or sustained twitching of the muscles innervated by that nerve. Although HFS is not fatal, it often leads to social embarrassment and interference with vision from involuntary eye closure leading to functional disability [1–3]. It is characterized by involuntary clonic or tonic contractions of the facial expression muscles, usually unilateral and rarely bilateral, beginning in the periorbital musculature usually starting in the low eyelid and progressing to the perioral, platysma and other facial expression muscles [4]. The symptoms of HFS are aggravated by stress, chewing, speech, light, cold stimuli and fatigue while relieved by quiet. Unlike other movement disorders, contractions persist in sleep which may add to the morbidity of the condition by predisposing the affected individual to disturbed sleep and insomnia. One recent research using facial electromyogram (EMG) and electrocardiogram (ECG) overnight indicated that wake or light sleep stages were more often accompanied by HFS [5]. The percentage of abnormal contraction of facial muscles in sleep was as high as 80% which influences sleep quality. To our knowledge, there were no reports on how HFS manifests under disturbance of consciousness. Here, we report a case of primary HFS in which the patient's symptoms persisted in a coma.

## Case presentation

**Fig. 1:**
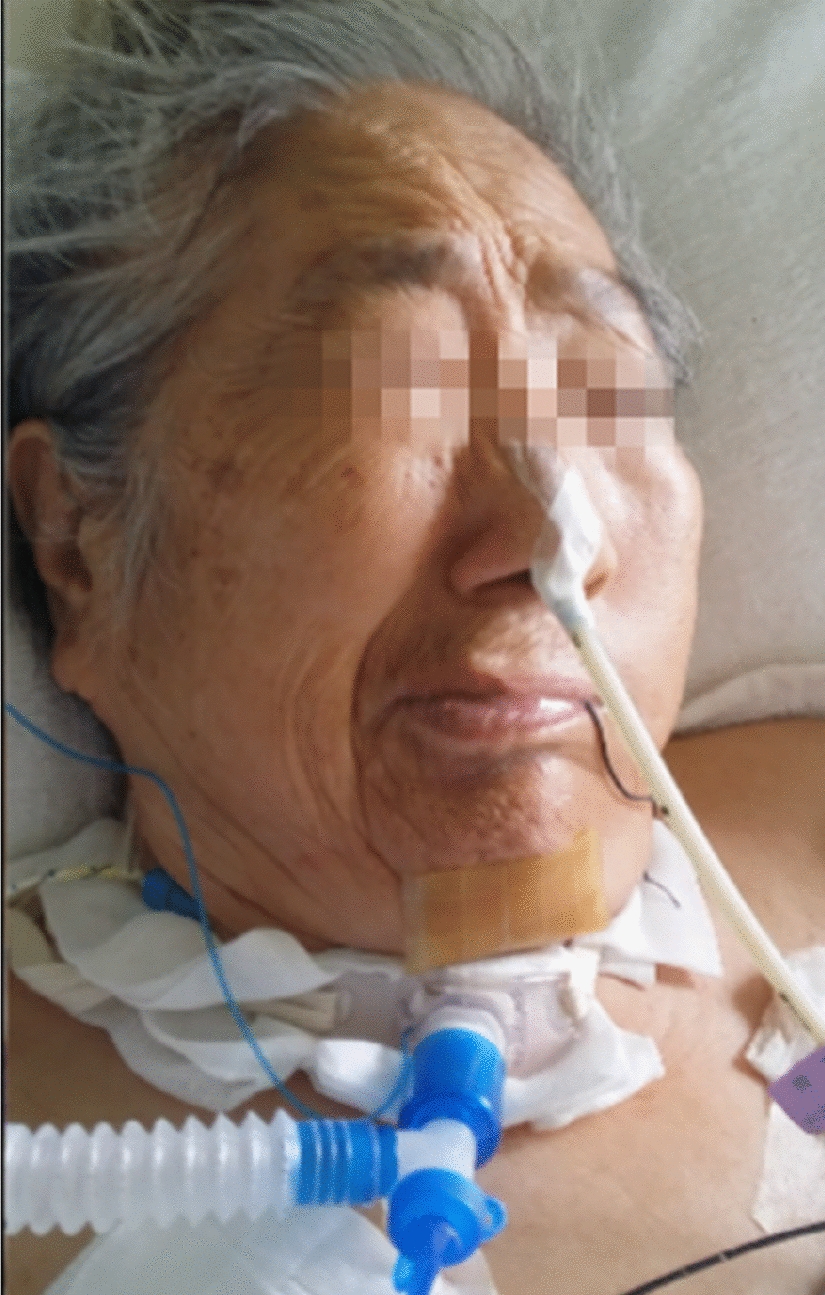
The patient was in a coma and tracheotomy with a life-support machine. Contractions of right-side muscles of facial expression existed. She also presented with paradoxical rising of the eyebrow as the orbicularis oculi contracted which is ‘the other Babinski sign’

A 74-year-old female with right-sided primary HFS for 20 years and no other medical conditions had received botulinum toxin injections in our hospital. Unfortunately, she was carried to emergency department after traumatic right pneumothorax by accident. During the emergency treatment, she lost consciousness due to simultaneous cardiac arrest and respiratory arrest. Emergency physicians immediately performed cardiopulmonary resuscitation (CPR), followed by tracheal intubation and closed chest drainage. After about 3 min, her heartbeat resumed. However, her consciousness did not return to normal. She was then admitted to the emergency intensive care unit for further treatment, including a subsequent tracheotomy. During her hospitalization, she was in a coma with stable vital signs and persisting symptoms of HFS. Thus, a multidisciplinary consultation was request to identify whether it was focal cortical seizures involving the right facial muscles. Though she was under coma status and on a life-support machine, the physical examination revealed right-side facial musculature involved in involuntary movement (Fig. 1). Transient involuntary clonic or tonic contractions have also been observed, especially when external stimuli such as oral care can exacerbate the symptoms. Her orbicularis oculi and eyelids contracted, internal part of the frontalis contracted and ipsilateral eyebrow rose (Additional file 1: Video S1). A combination of relevant data, including her past history, clinical presentation and a negative computed tomography (CT) scan of the head, led to a diagnosis of right-sided HFS. As the symptoms of HFS are not life-threatening, the use of anticonvulsants is unnecessary. The treatment should focus on life support and arousal.

## Discussion and conclusions

One important diagnostic clue of HFS is called the ‘Babinski-2 sign’, ‘the other Babinski sign’ or ‘brow lift sign’ and was firstly described by Babinski in 1905 [6–9]. It refers to the synchrony of the temporal and zygomatic branches of the facial nerve resulting in simultaneous contraction of the frontalis and orbicularis oculi muscles, which is not seen in other facial spasm disorders such as blepharospasm and hemifacial seizures. While the sensitivity is 86%, the specificity is nearly 100% for HFS, and among trained professionals, there is a high consensus in identification of this sign which cannot be voluntarily reproduced by an individual [10].

For the most part, we observe HFS in awakenings. Researches involving 16 and 12 patients, respectively, using polysomnography (PSG) indicate that HFS does not disappear in sleep but may decrease in strength of muscle contraction significantly compared to the waking period [11, 12]. In addition, as we have reported, symptoms of HFS persist in patients with disturbances of consciousness.

Although HFS is recognized as a disorder by movement disorder specialists, similar disorders can sometimes mimic its appearance, such as partial motor seizures. For the layperson, it is crucial to seek a multidisciplinary consultation to differentiate the diagnosis of focal cortical epilepsy involving the face and to obtain a correct diagnosis [13]. It is important to distinguish between the two conditions, particularly in the emergency department and intensive care unit, as seizures are common and may be inadvertently misdiagnosed or inappropriately diagnosed or treated [14, 15]. In patients with isolated facial movements of unknown or unclear etiology, EEG and multidisciplinary consultation should be strongly considered in the event of diagnostic confusion [16]. However, due to the critical condition of our patient, who was on life support, it was impractical to perform an EEG and facial EMG to detect any abnormal muscle response. HFS can be treated surgically or with oral medications, such as benzodiazepines, baclofen, and anticonvulsants if possible, but botulinum toxin injections are generally considered to be the treatment of first choice [17]. Herein symptoms of HFS were not life-threatening and anticonvulsants were unnecessary [18].

In this case, we have demonstrated the presentation of HFS in the presence of impaired consciousness, and multidisciplinary consultation made the diagnosis and subsequent treatment appropriate.

## Supplementary Information

**Additional file 1: Video S1.** The patient was in a coma and tracheotomy with a life-support machine in emergency department. Contractions of right-side muscles of facial expression existed. She also presented with paradoxical rising of the eyebrow as the orbicularis oculi contracted which is ‘the other Babinski sign’. Meanwhile, slight asymmetry of the eyebrows in spasm intervals existed.

## Data Availability

Not applicable.

## Abbreviation

HFS: Hemifacial spasm

## References

[1] Duarte GS, Rodrigues FB, Castelão M, Marques RE, Ferreira J, Sampaio C (2020). Botulinum toxin type A therapy for hemifacial spasm. Cochrane Database Syst Rev.

[2] Xiao L, Pan L, Li B, Zhou Y, Pan Y, Zhang X (2018). Botulinum toxin therapy of hemifacial spasm: bilateral injections can reduce facial asymmetry. J Neurol.

[3] Jankovic J (2009). Peripherally induced movement disorders. Neurol Clin.

[4] Conte A, Falla M, Diana MC, Bologna M, Suppa A, Fabbrini A (2014). Spread of muscle spasms in hemifacial spasm. Mov Disord Clin Pract.

[5] Hamasaki T, Morioka M, Fujiwara K, Nakayama C, Harada M, Sakata K (2018). Is hemifacial spasm affected by changes in the heart rate? A study using heart rate variability analysis. Clin Neurophysiol.

[6] Stamey W, Jankovic J (2007). The other Babinski sign in hemifacial spasm. Neurology.

[7] Devoize JL (2011). Neurological picture. Hemifacial spasm in antique sculpture: interest in the 'other Babinski sign'. J Neurol Neurosurg Psychiatry.

[8] Pawlowski M, Gess B, Evers S (2013). The Babinski-2 sign in hemifacial spasm. Mov Disord.

[9] Varanda S, Rocha S, Rodrigues M, Machado Á, Carneiro G (2017). Role of the "other Babinski sign" in hyperkinetic facial disorders. J Neurol Sci.

[10] McDowell MM, Zhu X, Hughes MA, Sekula RF (2016). Facial spasms, but not hemifacial spasm: a case report and review of literature. Childs Nerv Syst.

[11] Montagna P, Imbriaco A, Zucconi M, Liguori R, Cirignotta F, Lugaresi E (1986). Hemifacial spasm in sleep. Neurology.

[12] Incirli SU, Yilmaz R, Akbostanci MC (2019). Hemifacial spasm in sleep—a polysomnographic study. J Clin Neurosci.

[13] Waugh J, Prabhu SP, Bourgeois B, Kothare S (2011). Clinical reasoning: a 16-year-old girl with fixed unilateral grimace. Neurology.

[14] Deluca C, Tommasi G, Moretto G, Fiaschi A, Tinazzi M (2008). Focal motor seizures mimicking hemifacial spasm. Parkinsonism Relat Disord.

[15] Sringean J, Dressler D, Bhidayasiri R (2019). More than hemifacial spasm? A case of unilateral facial spasms with systematic review of red flags. J Neurol Sci.

[16] Espay AJ, Schmithorst VJ, Szaflarski JP (2008). Chronic isolated hemifacial spasm as a manifestation of epilepsia partialis continua. Epilepsy Behav.

[17] Yang SS, Seet RC, Lim EC (2009). Action-induced hemifacial spasm and its resolution with botulinum toxin. Mov Disord.

[18] Baizabal-Carvallo JF, Jankovic J (2017). Distinguishing features of psychogenic (functional) versus organic hemifacial spasm. J Neurol.

